# Supplementary material for: A user-friendly machine-learning program to quantify stomatal features from fluorescence images
Source: bioRxiv. 2025 Nov 21:2025.11.20.689597. Preprint. [Version 1] doi: 10.1101/2025.11.20.689597 (PMC12667868; doi:10.1101/2025.11.20.689597)
Supplement: 1 [file NIHPP2025.11.20.689597v1-supplement-1.pdf]

bioRxiv preprint doi: https://doi.org/10.1101/2025.11.20.689597; this version posted November 21, 2025. The copyright holder for this preprint (which was not certified by peer review) is the author/funder, who has granted bioRxiv a license to display the preprint in perpetuity. It is made available under aCC-BY 4.0 International license.

Figure S1.

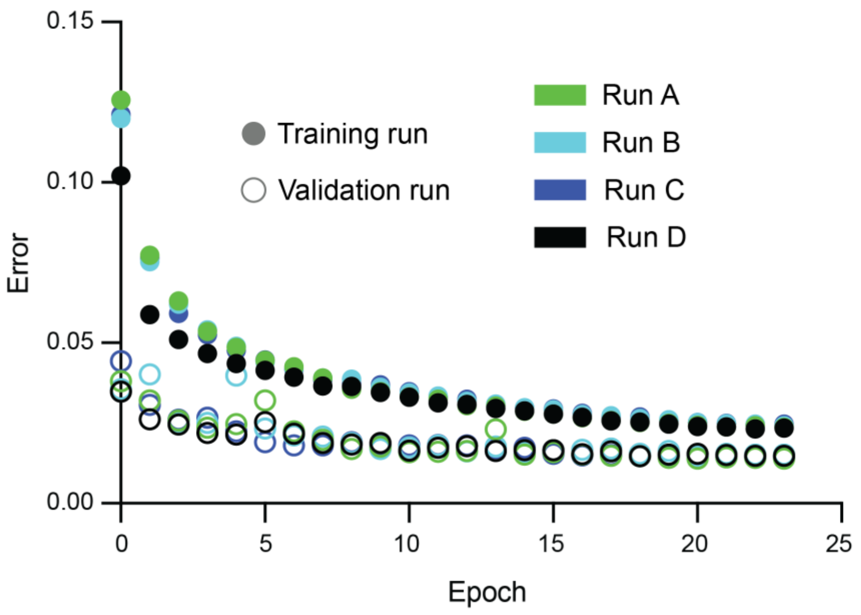

Figure S1. Results from QuickSpotter training cycles.
A graph showing a set of training cycles used to verify QuickSpotter's performance consistency. QuickSpotter's use of a non-deterministic data augmentation pipeline results in variable fitting performance, since no two input datasets for each epoch will be identical. Here, over four training/validation runs, divergent performance in the early epochs (0-5) converges to common values later in training (15-24). Training and validation runs sharing the same color occurred concurrently.

bioRxiv preprint doi: https://doi.org/10.1101/2025.11.20.689597; this version posted November 21, 2025. The copyright holder for this preprint (which was not certified by peer review) is the author/funder, who has granted bioRxiv a license to display the preprint in perpetuity. It is made available under aCC-BY 4.0 International license.

Figure S2.

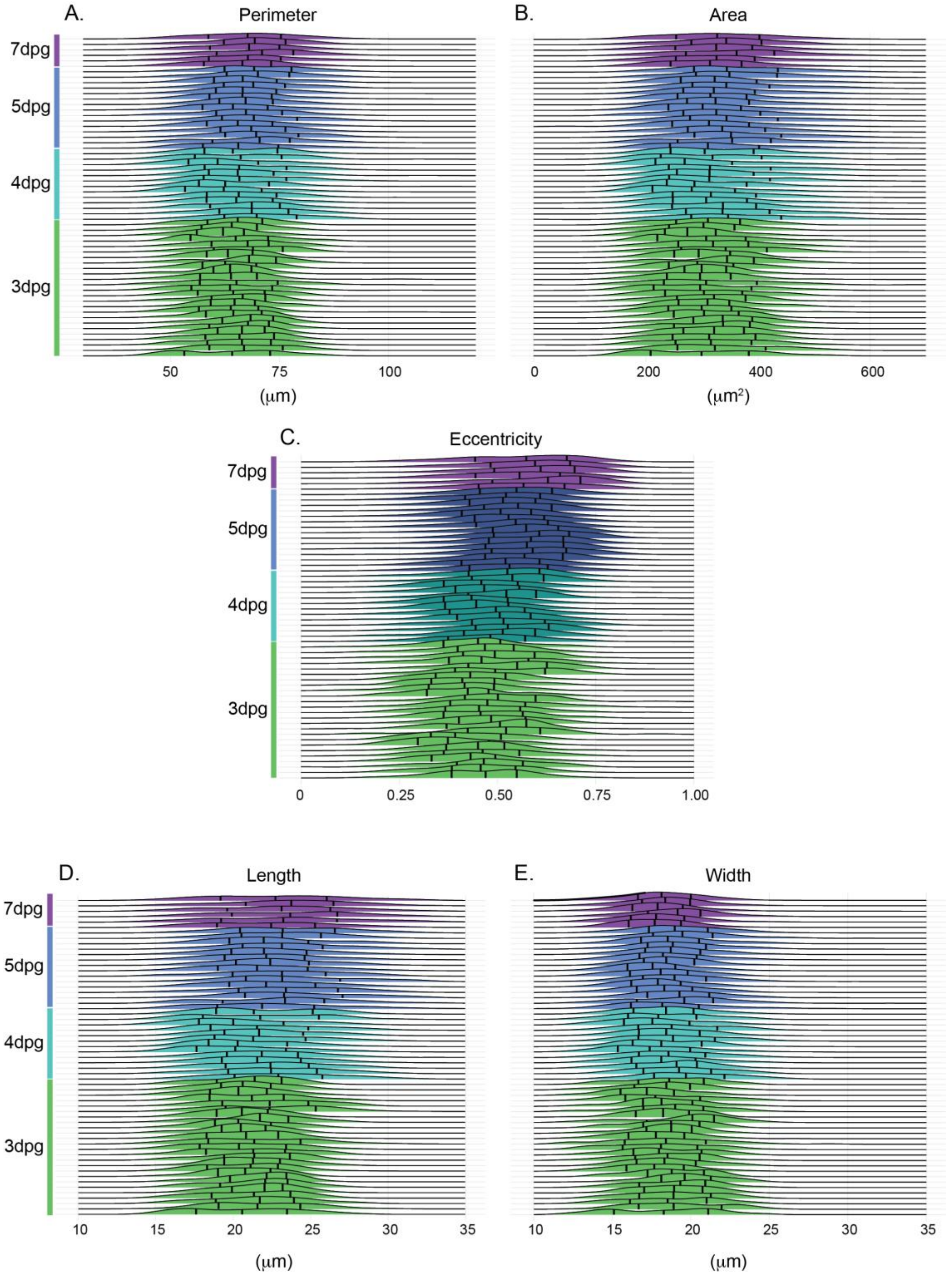

Figure S2. Stomatal morphology across cotyledon development.
Ridgeplots of stomatal (A) perimeter, (B) area, (C) eccentricity, (D) length, and (E) width at 3, 4, 5 and 7dpg. Each line shows the stomatal population from a single cotyledon.

bioRxiv preprint doi: https://doi.org/10.1101/2025.11.20.689597; this version posted November 21, 2025. The copyright holder for this preprint (which was not certified by peer review) is the author/funder, who has granted bioRxiv a license to display the preprint in perpetuity. It is made available under aCC-BY 4.0 International license.

Supplemental Table 1. Statistics associated with stomatal characteristics across cotyledon development and following TOR inhibition. P-values for the pairwise comparisons between the indicated groups using Kruskal-Wallis tests followed by Dunn's tests for the post-hoc analysis.

**Comparison**	**Stomatal count**	**Stomatal density**	**Stomatal area**	**Stomatal perimeter**	**Eccentricity**
3 vs. 4dpg	0.00238	0.0000494	0.236	0.31	0.53
3 vs. 5dpg	0.000000118	0.0000159	0.0000104	0.00000471	0.00000174
3 vs. 7dpg	0.000000225	0.000000166	0.054	0.0108	0.0000169
4 vs. 5dpg	0.603	1	0.142	0.0742	0.0236
4 vs. 7dpg	0.0538	0.255	1	0.76	0.0104
5 vs. 7dpg	1	0.24	1	1	1
DMSO vs. 100nM AZD	1	1	0.419	0.446	1
DMSO vs. 250nM AZD	1	1	0.0000101	0.000068	1
DMSO vs. 1μM AZD	0.00000708	0.00000382	0.00000157	0.00000341	1
100nM vs. 250nM AZD	1	1	0.0495	0.0491	1
100nM vs. 1μM AZD	0.00000389	0.000159	0.00794	0.0119	1
250nM vs. 1μM AZD	0.00308	0.000228	1	1	1
